# Contamination of Zearalenone from China in 2019 by a Visual and Digitized Immunochromatographic Assay

**DOI:** 10.3390/toxins12080521

**Published:** 2020-08-14

**Authors:** Xia Hong, Yuhao Mao, Chuqin Yang, Zhenjiang Liu, Ming Li, Daolin Du

**Affiliations:** Institute of Environmental Health and Ecological Security, School of the Environment and Safety Engineering, Jiangsu University, Xuefu Road 301, Zhenjiang 212013, China; hongxiaujs@hotmail.com (X.H.); maoyuhao7@gmail.com (Y.M.); yangchuqin18@gmail.com (C.Y.); lzj1984@ujs.edu.cn (Z.L.)

**Keywords:** zearalenone, immunochromatographic assay, semi-quantification, quantification

## Abstract

Zearalenone (ZEN) is a prevalent mycotoxin that needs intensive monitoring. A semi-quantitative and quantitative immunochromatographic assay (ICA) was assembled for investigating ZEN contamination in 187 samples of cereal and their products from China in 2019. The semi-quantitative detection model had a limit of detection (LOD) of 0.50 ng/mL with visual judgment and could be completely inhibited within 5 min at 3.0 ng/mL ZEN. The quantitative detection model had a lower LOD of 0.25 ng/mL, and ZEN could be accurately and digitally detected from 0.25–4.0 ng/mL. The ICA method had a high sensitivity, specificity, and accuracy for on-site ZEN detection. For investigation of the authentic samples, the ZEN-positive rate was 62.6%, and the ZEN-positive levels ranged from 2.7 to 867.0 ng/g, with an average ZEN-positive level being 85.0 ng/g. Of the ZEN-positive samples, 6.0% exceeded the values of the limit levels. The ZEN-positive samples were confirmed to be highly correlated using LC-MS/MS (R^2^ = 0.9794). This study could provide an efficiency and accuracy approach for ZEN in order to achieve visual and digitized on-site investigation. This significant information about the ZEN contamination levels might contribute to monitoring mycotoxin occurrence and for ensuring food safety.

## 1. Introduction

Zearalenone (ZEN) is a common secondary metabolite from the *Fusarium* species, which has become one of the most widespread mycotoxins and has caused substantial economic losses to grains around the world [1,2]. ZEN exposure could cause the genotoxic, hepatoxic, immunotoxic, and even estrogenic effects [3,4,5,6]. Thus, ZEN is categorized as a class III carcinogen [7]. Recent investigations into mycotoxin contamination from all over the world found that a high percentage of ZEN contamination exists in cereals and animal feed [8,9,10]. To better monitor ZEN contamination and maintain human health, the maximum limits (MLs) of ZEN in unprocessed cereals and unprocessed maize have been regulated by the European Commission (EC) to be no more than 100 ng/g and 350 ng/g, respectively [11]. The Expert Committee of both the Food and Agriculture Organization (FAO) and the World Health Organization (WHO) had set a provisional maximal tolerable daily intake of 0.5 ng/g of body weight for ZEN [12]. The China National Standard regulates that ZEN should be no more than 60 ng/g in wheat, wheat flour, corn, and corn flour [13].

High performance liquid chromatography (HPLC) [14], HPLC-tandem mass spectrometry (HPLC-MS) [15], and immunoassays [16,17] are used for detecting ZEN. Compared with the referenced HPLC and HPLC-MS methods, immunoassays have the characteristics of rapidity, being low cost, and being able to achieve high throughput screening on-site for a large number of samples [18,19,20,21]. Of the variety of immunoassays, the immunochromatographic assay (ICA) has attracted more attention. It is widely used for detecting contamination, because of its outstanding characteristics of simplicity, readability, and portability [22,23].

ICA methods have been developed and applied for ZEN detection. An ICA strip has been used for the rapid detection of ZEN in wheat from Jiangsu, China, with a limit of detection (LOD) of 50 ng/mL, which was applied in 202 real wheat samples [24]. The ICA method based on the quantum dot nanobead and biotin-streptavidin system for the determination of aflatoxin B_1_ (AFB_1_) and ZEN was developed, which improved the sensitivity of ZEN detection to 59.15 pg/mL [25]. A quantum dot microbead based fluorometric lateral flow ICA was developed for the simultaneous detection of AFB_1_, deoxynivalenol (DON), and ZEN, and the LOD reached 1.92 ng/g for ZEN in the real cereal samples [26]. Furthermore, amorphous carbon nanoparticles, aptamer, dyed latex microspheres, and other novel materials have been used to improve the performance of ICA for detecting ZEN [27,28,29]. The above-mentioned ICA methods for ZEN promoted detection sensitivity, for which it also showed the characteristics of convenience, rapidity, economy, visual detection on-site, and could even get accurate levels of ZEN contamination using the quantitative approach.

**Figure 1:**
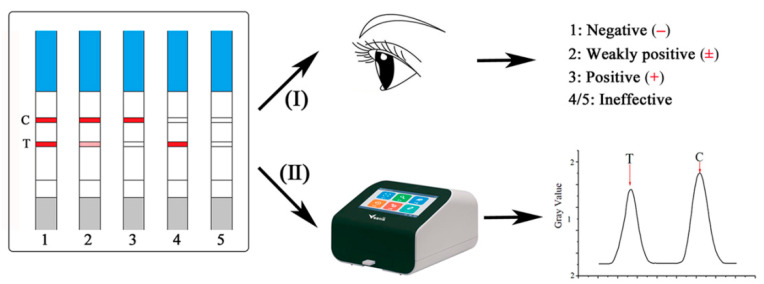
Two models of result judgment for the immunochromatographic assay (ICA).

Given the high ZEN contamination rate and the large number of samples to be examined, a higher performance detection method for ZEN was developed and improved in this study. For this purpose, a practical ICA based on two judgment models for the semi-quantitative detection and quantitative detection of ZEN was developed and applied in authentic cereals and feeds (Figure 1). Combining the naked eye and strip reader, the ICA could quickly achieve the visualization and digitalization for ZEN detection, while improving the detection sensitivity and providing an alternative detection method for ZEN. The proposed ICA method was applied to detect the ZEN contamination levels in 187 samples of cereal and their products from China in 2019. Then, the referenced LC-MS/MS was used to verify the accuracy of the ICA method.

## 2. Results and Discussion

### 2.1. Identification of the Gold Nanoparticles

**Figure 2:**
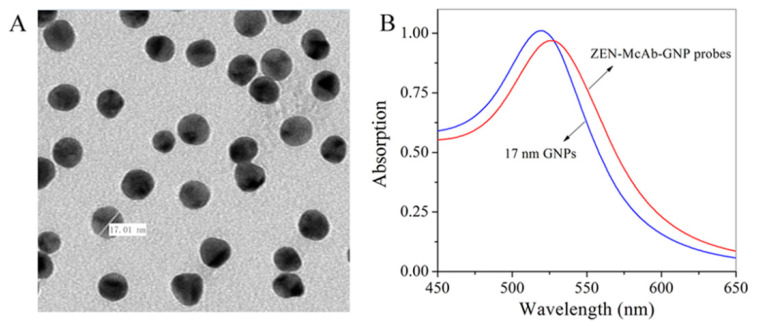
The transmission electron micrograph image for the gold nanoparticles (GNPs) (**A**) and the UV/vis spectra for the GNPs and zearalenone (ZEN)-monoclonal antibody (McAb)-GNP probes (**B**).

The image of the transmission electron micrograph showed that the prepared colloidal gold nanoparticles (GNPs) were about 17 nm and the particles exhibited a uniform distribution (Figure 2A). The ultraviolet–visible spectra (UV/vis) for the GNPs solution had a smooth protruding peak at 518 nm. Qualitative differences were found between the UV/vis spectra of the ZEN-monoclonal antibody (McAb)-GNP probes and GNPs (Figure 2B). The ZEN-McAb-GNP probes showed a smooth protruding peak at 527 nm, indicating that the GNPs had been successfully coupled in ZEN-McAb to produce probes. Further studies of the ICA evaluation and application also demonstrated the availability and excellent performance of the prepared GNPs.

### 2.2. Key Parameters for the ICA

It was a key procedure to optimize the parameters for the preparation of the ZEN-McAb-GNP probes and the ICA strips, which could be beneficial for improving the sensitivity of the ICA method. The evaluation criterion was based on using fewer ZEN in order to reduce the red color intensity in the T line and for maintaining the effectiveness of ICA. The optimal parameters for the ICA are shown in Table 1. For preparing the ZEN-McAb-GNP probes, 2.3 μL of 0.2 mol/L K_2_CO_3_ in an aqueous solution, 3.0 μL of a 1.0 mg/mL ZEN-McAb in a 10 mmol/L PB buffer, and 100 μL of 10% bovine serum albumin (BSA) in purified water were used in 1 mL of GNPs. For spraying a 30 cm of nitrocellulose (NC) membrane, the 0.5 μL of 13.1 mg/mL goat anti-mouse IgG (GAM-IgG) and 1.0 μL of 8.8 mg/mL ZEN-antigen in 30 μL of 10 mmol/L of phosphate buffered saline (PBS buffer) were immobilized on the C line and T line, respectively.

### 2.3. Semi-Quantitative Detection of the ICA

**Figure 3:**
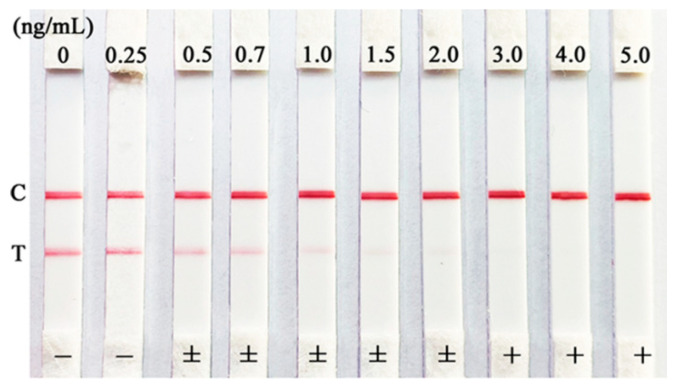
The semi-quantitative detection of ICA for the series of ZEN standards.

A series of ZEN standards (0, 0.25, 0.5, 0.7, 1.0, 1.5, 2.0, 3.0, 4.0, and 5.0 ng/mL in 20% methanol of 10 mmol/L of PBS buffer) were detected by the developed ICA. With the increasing in ZEN standards, the red color intensities were in a gradually decreasing process, and eventually disappeared for the T lines (Figure 3). The red color intensities for the C lines always existed to prove the effectivity of the ICA. It was observed that a significant reduction in the red color in the T line was shown in 0.50 ng/mL of ZEN. Thus, the semi-quantitative LOD of the ICA could be established as 0.50 ng/mL. The red color intensities eventually disappeared in the T line when the concentration of ZEN exceeded 3.0 ng/mL. These results of the ICA judged by the naked eye indicated that the visual detection of the ZEN levels could be in three intervals: <0.50 ng/mL (−, negative), 0.50 ng/mL ≤ZEN concentration <3.0 ng/mL (±, weakly positive), and ≥3.0 ng/mL (+, positive).

### 2.4. Quantitative Detection of the ICA

**Figure 4:**
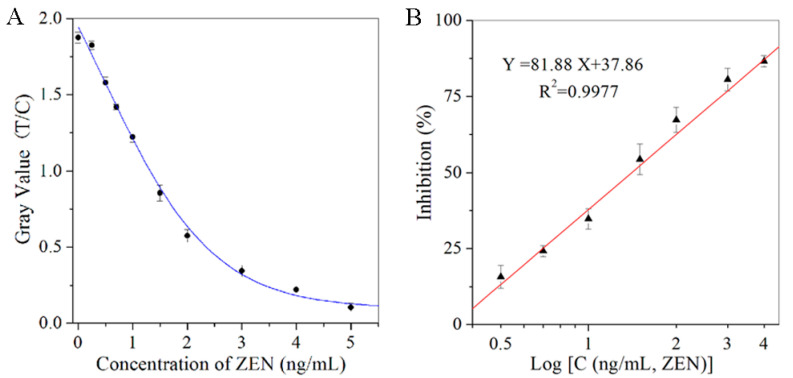
The quantitative detection of ICA for the series of ZEN standards. (**A**) The corresponding relationship between the gray value (T/C) and the concentration of ZEN (ng/mL); (**B**) The linear relationship between the inhibition (%) and the logarithm of the ZEN concentrations (ng/mL).

The strips of ICA were digitally detected by the strip reader, and the gray values of the T lines were plotted against the concentrations of ZEN in order to get a reduction curve (Figure 4A). This showed a good linear relationship between the inhibition ratios of the gray values in the T lines and the logarithm of the ZEN concentrations (Figure 4B; Y = 81.88X + 37.86, R^2^ = 0.9977). In this case, ZEN could achieve linear detection in the range of 0.25 to 4.0 ng/mL, and the quantitative LOD could be defined as 0.25 ng/mL (Table 1). With the semi-quantitative and quantitative LOD of the ICA all below the MLs for ZEN, the sensitivity of the developed ICA was satisfied for detecting ZEN.

Comparing the developed ICA with the enzyme-linked immunosorbent assay (ELISA) for ZEN, ICA showed more convenient and rapid operation steps for on-site detection, which could achieve ZEN detection within 5 min using one step. Two models of judgment using the naked eye and strip reader could allow for the ICA detection to be more flexible. The digitized detection of ICA showed a lower LOD and a wider detection range than the visual detection. It was close to the sensitivity of the enzyme-linked immunosorbent assay (ELISA; Table S1 and Figure S1). Moreover, the digitized detection of ICA could obtain accurate ZEN levels in order to realize quantitative detection. It was suggested that the visual and digitized detection of ICA for ZEN contamination could be selected as needed or used simultaneously in order to achieve semi-quantitative and quantitative detection for on-site testing.

### 2.5. Specificity of the ICA

**Figure 5:**
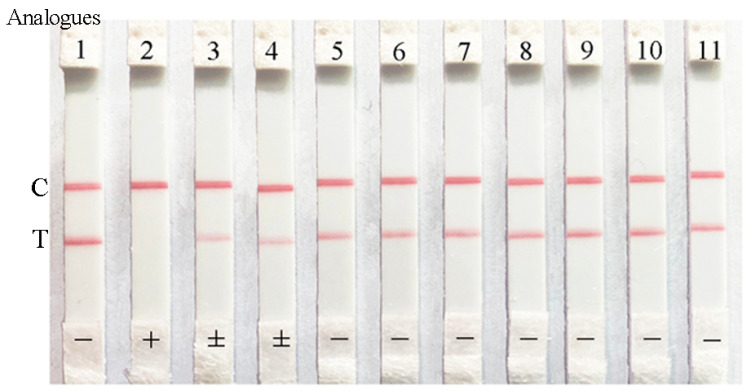
The cross-reactivity tests of the ICA for ZEN detection. The analogs were 5 ng/mL. (1) negative; (2) ZEN; (3) α-Zearalenol; (4) β-Zearalenol; (5) α-Zearalanol; (6) β-Zearalanol; (7) Zeranol; (8) Aflatoxin B_1_; (9) Deoxynivalenol; (10) Fumonisin B1; (11) Ochratoxin A.

The cross-reactivity tests (CR; %) of the ICA for the analogs of ZEN and the common mycotoxins were carried out in order to assess the specificity of the developed ICA method. It could be observed that ZEN made the red color completely disappear, and α-Zearalenol or β-Zearalenol left the red color markedly diminished, while other compounds caused the T lines to be obviously weaker than that the C line when the compounds were 5 ng/mL in the sample solutions (Figure 5). Further study showed that the CRs of the ICA method for α-Zearalenol, β-Zearalenol, α-Zearalanol, β-Zearalanol, and Zeranol were 13.0%, 10.5%, 0.5%, 0.48%, and 0.35%, respectively, which indicated that the ICA method had a slight specificity for the ZEN analogs. At the same time, the CRs of the ICA method for common mycotoxins were less than 0.1%, which indicated that the ICA method could not identify other mycotoxins. Thus, the developed ICA method had a high specificity for ZEN detection.

### 2.6. Stability of the ICA

The effective test and the sensitivity test of the ICA for ZEN over 3 months is shown in Figure S2. During the 3 months of storage life and interval sampling detection, the red color intensity of the T lines and C lines were kept clear and had distinct effects. Meanwhile, the T lines completely disappeared at 5 ng/mL of ZEN standard, which meant that the sensitivity of the ICA was well represented in this storage life. This indicates that the developed ICA for ZEN had a desirable stability, and the useful life could be at least 3 months.

### 2.7. Accuracy and Precision of the ICA

After the steps of pretreatment and being diluted at 10:1, the matrix effect for the spiked samples effectively decreased. The free of ZEN and 3 ng/g of ZEN in the spiked samples were judged to be negative (−), and 20 ng/g of ZEN was weakly positive (±) and 50 ng/g of ZEN was positive (+) using the ICA detection using naked eye, which we achieved by visualization and semi-quantitative detection. Then, the spiked sample extracts were quantitatively detected by the ICA coupling strip reader in order to achieve digitization with ZEN that ranged from 2.6 to 59.3 ng/g. The quantitative recoveries of the spiked samples were between 86.7 to 118.6%, with the standard deviation (SD) being from 3.1% to 6.2%. These results indicate that the accuracy and precision of the ICA were desirable for the semi-quantitative and quantitative detection for ZEN contamination in the cereal and feed samples (Table 2).

### 2.8. Investigation of Authentic Samples by the ICA and LC-MS/MS

Using the ICA method, the ZEN contamination in the authentic samples was visually judged following the digitized detection. The results of the semi-quantitative and quantitative detection by the two models of visualization and digitization for ICA were in good agreement. Furthermore, the digitized detection of ICA could obtain accurate ZEN levels in the authentic samples, and some negative samples might have also detected ZEN contamination, which could further expand the detection ability and sensitivity of the developed ICA method. A total of 187 cereals and its product samples were investigated for ZEN contamination from China in 2019. The results of the ZEN levels using the quantitative ICA are shown in Table 3. The ZEN-positive rate was 62.6% in 117 out of the 187 total samples, which detected positivity in 28 out of 40 corn samples, 7 out of 19 wheat samples, 17 out of 39 wheat flour samples, 39 out of 49 cereal product samples, and 26 out of 40 feed samples. From the entire sample, the ZEN-positive levels ranged from 2.7 to 867.0 ng/g, with an average ZEN-positive level being 85.0 ng/g.

**Figure 6:**
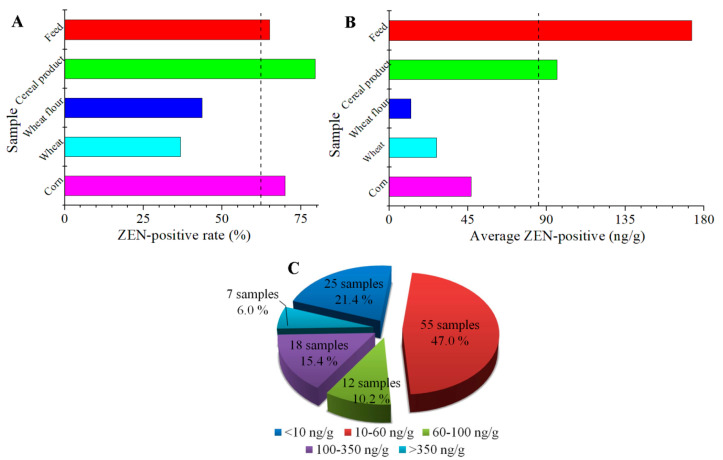
The distribution of ZEN-positive rate (**A**), average ZEN-positive level (**B**) and ZEN-positive sample at different levels (**C**) from China in 2019.

The highest ZEN-positive rate was found in the cereal product samples (79.6%), which, along with the corn samples (70.0%) and the feed samples (65.0%), had above the average ZEN-positive rate (62.6%; Figure 6A). The average ZEN-positive levels for the cereal product and feed samples reached 95.9 ng/g and 173.0 ng/g, which were higher than the average ZEN-positive level of all of the samples (85.0 ng/g; Figure 6B). The lowest average ZEN-positive level was detected in the wheat flour samples, thus demonstrating the lower ZEN-exposure risk for wheat flour. To make a further assessment of the ZEN-exposure levels from China in 2019, the distribution of the ZEN-positive sample and ZEN-positive rate at different levels is shown in Figure 6C. The ZEN-positive levels were classified into five levels, namely, <10 ng/g, 10–60 ng/g, 60–100 ng/g, 100–350 ng/g, and >350 ng/g, according to the MLs from European Commission and the China National Standard. The ZEN-positive levels of 68.4% with 80 out of 117 ZEN-positive samples were lower than the ML value of China (60 ng/g), while 94% of samples had ZEN-positive levels less than the European Commission regulation of 350 ng/g. However, it is noteworthy that 6.0% in 117 of the ZEN-positive samples showed ZEN levels more than 350 ng/g, exceeding the ML value of China and the European Commission. In addition, the highest ZEN-positive level was detected as 867.0 ng/g in a feed sample, which had been 14-fold and 2.5-fold higher than the ML values for China and the European Commission, respectively. These results indicate that ZEN contamination was a commonly occurring problem for the authentic cereals and their product samples from China in 2019, and most of the contamination levels were within the bounds of the control, but some samples were seriously ZEN-positive and needed to be focused on in order to ensure food safety and human health.

The LC-MS/MS detection showed that ZEN contaminations in the positive samples ranged from 3.2 to 761.7 ng/g (Table S2). The large correlation of results from the quantitative ICA method and LC-MS/MS method for detecting the ZEN in the authentic samples was R^2^ = 0.9794 (Y = 0.94X + 4.8442; Figure S3), which further verified the reliability and accuracy of the proposed ICA method.

## 3. Conclusions

To further enhance the detection ability and ensure food safety, a semi-quantitative and a quantitative detection method of ICA were successfully established and used for detecting ZEN in cereal and feed samples. The results of the ICA could be judged visually by the naked eye or with a digitized strip reader within 5 min. The visual LOD for ZEN was 0.50 ng/mL using the semi-quantitative ICA. The quantitative ICA had a lower LOD of 0.25 ng/mL, and a wider detection range, which could obtain accurate ZEN levels. The powerful detection capability of the developed ICA was demonstrated by the evaluation of its sensitivity, specificity, stability, accuracy, and precision. The ICA could dramatically shorten the analytical procedure and the overall detection time when compared with the micro-well based ELISA or chromatographic-based HPLC method. A total of 187 samples of authentic cereals and their products from China in 2019 were investigated for ZEN contamination by both the developed ICA and the referenced LC-MS/MS, in order to demonstrate the reliability of the proposed ZEN detection method. The ZEN-positive rate was 62.6%, and the ZEN-positive levels ranged from 2.7 to 867.0 ng/g, with an average ZEN-positive level being 85.0 ng/g. The highest ZEN-positive level was detected as 867.0 ng/g in a feed sample. It is noteworthy that the ZEN contamination levels of 6.0% in 117 ZEN-positive samples exceeded the ML value of the China and European Commission. The results of this investigation suggest that ZEN contamination in China occurred widely and had a high detection rate. The efficiency and accuracy of the ZEN detection could have further improvement, and the study could provide an alternative approach and valuable information about ZEN contamination in China.

## 4. Materials and Methods

### 4.1. Reagents and Materials

ZEN, its analogs, and common mycotoxin standards were purchased from Pribolab Pte. Ltd. (Biopolis, Singapore). The BSA and GAM-IgG were supplied by Sigma-Aldrich (St. Louis, MO, USA). The trisodium citrate, HAuCl_4_, and K_2_CO_3_ were bought from Aladdin (Shanghai, China). The NC membranes were from Millipore (Bedford, MA, USA). The absorbent pad, sample pad, polyvinyl chloride (PVC) sheet, and glass-fiber conjugate pad were provided by Jiening Bio. Tech. Co., Ltd. (Shanghai, China). The ZEN antigen (ZEN-BSA) and McAb against ZEN (ZEN-McAb) were supplied by Biosco Biological Tech. Co., Ltd. (Dalian, China). The other reagents were all of analytical grade.

The XYZ3030 dispense platform (Kinbio Tech. Co., Ltd., Shanghai, China) and the HGS201 automatic programmable cutter (Hangzhou Autokun Tech. Co., Ltd., Hangzhou, China) were used in the preparation of the ICA strips. The Tecnai 12 transmission electron microscope (Philips, Eindhoven, Netherlands) was used to scan the diameter and shape of the nanoparticles. The Milli-Q purification system (Millipore, Bedford, MA, USA) was used for preparing the purified water. The TG16-WS high-speed centrifuge was used for centrifuging (Cence Co., Changsha, China). The vortex mixer was provided by North TZ-Biotech Develop. Co., Ltd. (Beijing, China). The results of the ICA for detecting ZEN were confirmed with an Agilent 1200-6460 LC-MS/MS equipped with electrospray ionization (Agilent, Wilmington, DE, USA). The results of the ICA were digitized on an HG-1721 strip reader (Vict Tech. Co., Ltd., Suzhou, China).

### 4.2. Preparation of the Nanoparticles

The GNPs were prepared using the reducing HAuCl_4_ method with trisodium citrate. Briefly, one milliliter of 1% HAuCl_4_ aqueous solution was added to 99 mL of boiling threefold-distilled water. Then, 1.5 mL of 1% trisodium citrate was quickly added to the above solution under the boiling and stirring condition. The mixed reaction solution was kept boiling for 5 min in order to let the GNPs develop after the color of solution changed from deep black into brilliant wine red. Then, the prepared GNPs were cooled down and stored at 4 °C. Finally, the diameters and dispersion of the GNPs were evaluated through a transmission electron microscope.

### 4.3. Preparation of the ZEN-McAb-GNP Probes

The ZEN-McAb-GNP probes were the essential biosensor materials for reflecting the detection results, which were prepared as per the previously reported method [30,31]. Firstly, appropriate dosages of 0.2 mol/L K_2_CO_3_ of an aqueous solution were used to adjust the GNPs solution (1 mL) to pH 8.5. Next, the 3.0 μL diluted ZEN-McAb was coupled with the GNPs at room temperature for 0.5 h after mixing well. Then, the BSA in purified water was used to block the nonspecific surface for 0.5 h. After removing the weak particles at low speed centrifugation, the reaction solution was separated through centrifugation in 8000 r/min at 4 °C for 15 min. Finally, the sediment resulted in the ZEN-McAb-GNP probes, which were redissolved in a 10 mmol/L Tris buffer containing a stabilizer and were stored at 4 °C.

### 4.4. Preparation of the ICA Strip

To prepare the ICA strip for ZEN detection, the various biochemical reagents were immobilized on the corresponding locations. Then, 30 μL of the diluted GAM-IgG or ZEN-antigen were uniformly sprayed on the NC membrane with 5 mm parallel spacing, and were dried for 5 h at 60 °C, which were the control line (C line) and test line (T line), respectively. The prepared ZEN-McAb-GNP probes were also uniformly sprayed on the glass-fiber conjugate pad and dried for 1 h at 37 °C. The assembly of the ICA strip for ZEN was the same as the previous technological process. The immobilized sample pad, glass-fiber conjugate pad, NC membrane, and absorbent pad were pasted onto a 30 cm PVC sheet. Then, the assembled pad was cut into a 4 mm wide ICA strip and stored in dry and dark conditions.

### 4.5. Procedure and Judgment of the ICA

When using the prepared ICA to detect ZEN, the ZEN standard solution or sample extraction solution (100 μL) was put onto the sample pad of the ICA strip. The ICA detection could be finished after 5 min under the capillary action and immune reaction. At that moment, the detection results of the red color intensity would be reflected on the T line and C line, which were inversely proportional with the ZEN levels in the detection solutions. The depth of the red color intensity could be evaluated by two models—the naked eye and strip reader—as shown in Figure 1. Using model I, the ICA results could be judged as the following four cases: (1) negative (−), the ZEN was less than the LOD or free; (2) weakly positive (±), the ZEN was more than the LOD but lower than the concentration of the complete inhibition of the immune reaction; (3) positive (+), the ZEN was above the concentration of the complete inhibition of the immune reaction; and (4) ineffective, the ICA strip was not working. Using model II, the ICA results could be detected digitally. The gray values of the T lines could be obtained, and the inhibition ratios could be used to establish a linear relationship with the concentrations of ZEN levels using model II.

### 4.6. Optimization of the ICA

The optimizations of the biochemical reagent dosages were essential for developing a sensitive and desirable ICA method for ZEN detection [32]. The key parameters were evaluated to improve the performance and sensitivity of the ICA, such as the dosage of K_2_CO_3_ to adjust the pH value; BSA to block the nonspecific site at the surface of the GNPs; and the concentrations of ZEN-McAb, ZEN-antigen, and GAM-IgG to prepare the ICA strips. During the optimization process, the sensitivity of detection and clear judgment of the red color intensity by the naked eye should be focused on and selected.

### 4.7. Evaluation of the ICA

Under the optimized vital parameters, the sensitivity of the ICA for ZEN would be improved. The two models for judging the results of the ICA could obtain semi-quantitative and quantitative detection. The corresponding minimum ZEN concentration, which could significantly reduce the red color in the T line compared with the reference, was defined as the semi-quantitative LOD. In the digitized ICA, the minimum ZEN concentration of the linear range was determined as the quantitative LOD. In addition, the cross-reactivity test and storage test were carried out to evaluate the specificity and stability of the developed ICA.

### 4.8. Detection of Spiked and Authentic Samples

The recovery test of the spiked samples and the verification of the authentic samples by LC-MS/MS were used to evaluate the accuracy and precision of the developed ICA for ZEN detection. A total of 187 authentic cereal and its product samples, including 40 corn, 19 wheat, 39 wheat flour, 49 cereal product, and 40 feed samples, were collected from China in 2019 (Tables S3 and S4). The cereal product samples were mainly corn gluten meal, corn gluten, corn germ, soybean meal, peanut meal, and rice bran meal. All of the authentic samples were homogenized and stored at −20 °C before the detection procedure.

For the recovery test, the cereal and feed samples (5.0 g), which had been confirmed to be free of ZEN contamination, were completely crushed and spiked the ZEN standard at 0, 3, 20, and 50 ng/g, and were stored for 2 h at room temperature. The methanol/water (12.5 mL, 1:1, *v*/*v*) was used as an extracting solution. Vortex blending was performed on the mixture for 5 min, and was then centrifuged for 10 min at 4000 rpm/min. The supernatant solution was diluted four-fold with a working buffer and was adjusted to pH 6–8 and detected by the developed ICA method. After 5 min, the strip of ICA flowed over the absorption pad, and the result was judged by the naked eye and the strip reader, respectively. The pretreatment and detection for the authentic cereal and its product samples were carried out in the same way as the above procedures. The correlation between the results of the ICA and LC-MS/MS was also evaluated.

To verify the reliability of the ICA method using the LC-MS/MS method [33,34], the completely crushed corn, wheat, or feed sample (5.0 g) was added in an extract solution of acetonitrile/water/formic acid (10 mL, 80: 19: 1, *v*/*v*/*v*). The mixture was mixed with an ultrasonic bath for 30 min, and then centrifuged at 4000 rpm/min for 5 min. The supernatant (10 mL) was transferred into another tube, followed by adding C_18_ (100 mg) and MgSO_4_ (200 mg). The mixture was vortexed for 3 min and centrifuged at 4000 rpm/min for 5 min. The supernatant (1 mL) was concentrated to dryness by nitrogen gas. The residue was redissolved in methanol/water (400 μL, 50:50, *v*/*v*) and filtered with a 0.22 μm nylon filter, then analyzed by the LC-MS/MS with an Agilent Zorbax SB C18 reverse-phase column (3.5 μm, 150 mm × 2.1 mm) with a column temperature of 40 °C. The mobile phase was the different volume ratio of the acetonitrile/water, and flowed on the gradient elution program at a flow rate of 0.2 mL/min. The analysis was performed by multi-reaction monitoring (MRM) technology, and the ion source was an electrospray ion (ESI). The capillary voltage was at 3.0 kV, and the argon collision pressure was 2.60 × 10^−4^ Pa. The mass-to-charge ratios of the ZEN parent ion, quantitative ion, and qualitative ion were 317.1, 174.9, and 273.9, respectively.

## Figures and Tables

**Table 1:** The optimization of key parameters for the preparation of ICA for ZEN.

Parameter	Concentration	Volume	Solvent
GAM-IgG	13.1 mg/mL	0.5 μL	10 mmol/L PBS (containing 0.15 mol/L NaCl, pH 7.4)
ZEN-antigen	8.8 mg/mL	1.0 μL	10 mmol/L PBS (containing 0.15 mol/L NaCl, pH 7.4)
ZEN-McAb	1.0 mg/mL	3.0 μL	10 mmol/L PB
BSA	10%	100 μL	Purified water
K_2_CO_3_	0.2 mol/L	2.3 μL	Purified water

Note: The concentrations and volumes of ZEN-McAb and K_2_CO_3_ in this table are used in 1 mL GNPs. Other parameters were used for preparing a 30 cm of NC membrane.

**Table 2:** The ZEN recoveries from using the ICA method in the spiked samples.

Sample	Spiked (ng/g)	Dilution Times	Visualization ^a^	Digitization (ng/g)	Quantitative Recovery ± SD (%, *n* = 5) ^b^
Corn	0	10	−	ND ^c^	ND
	3		−	3.4	113.3 ± 4.2
	20		±	18.6	93.0 ± 3.9
	50		+	59.3	118.6 ± 5.5
Wheat	0	10	−	ND	ND
	3		−	2.6	86.7 ± 4.6
	20		±	22.4	112.0 ± 3.7
	50		+	45.2	90.4 ± 5.8
Feed	0	10	−	ND	ND
	3		−	3.1	103.3 ± 4.9
	20		±	23.3	116.5 ± 6.2
	50		+	56.4	112.8 ± 3.1

^a^ The visual detection was based on the ZEN levels: <5 ng/g (−), 5 ng/g ≤ ZEN concentration < 30 ng/g (±); >30 ng/g (+). ^b^ Each value is the mean of three replicates. ^c^ Not detected.

**Table 3:** The investigation of the ZEN in the original samples using the quantitative ICA from China in 2019.

Item	All	Corn	Wheat	Wheat Flour	Cereal Product	Feed
Total samples	187	40	19	39	49	40
ZEN-positive samples	117	28	7	17	39	26
ZEN-positive rate (%)	62.6	70.0	36.8	43.6	79.6	65.0
Average ZEN-positive (ng/g)	85.0	46.8	27.1	12.4	95.9	173.0
ZEN-positive range (ng/g)	2.7–867.0	3.2–743.2	3.0–117.5	6.8–21.3	2.7–677.7	7.2–867.0

## Supplementary Materials

The following are available online at https://www.mdpi.com/2072-6651/12/8/521/s1, Table S1: The parameters of ELISA and ICA for ZEN, Table S2: The ZEN-positive levels in the authentic samples from China in 2019, Table S3: The information of the authentic samples from China in 2019, Table S4: The information of the authentic samples from China in 2019, Figure S1: The standard curve of ELISA for ZEN, Figure S2. The stability and sensitivity tests of the ICA strip for ZEN in the storage of 3 months, Figure S3. The correlation between the quantitative ICA and LC-MS/MS for ZEN in the authentic samples.
