# Supplementary material for: Contamination of Zearalenone from China in 2019 by a Visual and Digitized Immunochromatographic Assay
Source: Toxins (Basel). 2020 Aug 14;12(8):521. doi: 10.3390/toxins12080521 (PMC7472730; doi:10.3390/toxins12080521)
Supplement: Supplementary file 1 [file toxins-12-00521-s001.pdf]

Supplementary Materials: Contamination of Zearalenone from China in 2019 by a Visual and Digitized Immunochromatographic Assay

Xia Hong, Yuhao Mao, Chuqin Yang, Zhenjiang Liu, Ming Li and Daolin Du

Table S1. The parameters of ELISA and ICA for ZEN.

Immunoassay	Detection Method	LOD (ng/mL)	Detection Range (ng/mL)	Detection Step	Detection Time (min)
ELISA	Microplate reader	0.20	0.20–5.4	5	180
ICA	Naked eye	0.50	0.50–3.0	1	5
	Strip reader	0.25	0.25–4.0	1	5

Table S2. The ZEN-positive levels in the authentic samples from China in 2019.

Sample	Quantitative ICA (ng/g, *n* = 3)	LC-MS/MS (ng/g, *n* = 3)	Sample	Quantitative ICA (ng/g, *n* = 3)	LC-MS/MS (ng/g, *n* = 3)	Sample	Quantitative ICA (ng/g, *n* = 3)	LC-MS/MS (ng/g, *n* = 3)
Corn	3.2	2.9	Wheat flour	9.5	12.6	Corn gluten	76.9	96.6
Corn	3.5	2.8	Wheat flour	10.6	6.3	Soybean meal	84.2	76.1
Corn	6.2	5.7	Wheat flour	10.6	14.2	Soybean meal	120.3	129.6
Corn	7.4	9.9	Wheat flour	10.7	12.1	Peanut meal	121.2	128.4
Corn	7.5	9.0	Wheat flour	11.2	6.8	Peanut meal	132.9	139.3
Corn	7.9	8.0	Wheat flour	11.7	7.6	Rice bran meal	148.6	144.0
Corn	8.6	9.9	Wheat flour	12.4	11.6	Corn gluten meal	153.3	186.4
Corn	8.8	10.8	Wheat flour	13.9	9.0	Rice bran meal	208.6	249.5
Corn	8.9	9.8	Wheat flour	14.7	10.5	Rice bran meal	208.6	187.7
Corn	9.9	11.8	Wheat flour	14.7	16.2	Rice bran meal	229.1	143.7
Corn	10.0	9.7	Wheat flour	20.1	20.7	Peanut meal	263.1	279.2
Corn	10.2	12.8	Wheat flour	20.4	25.0	Corn gluten meal	477.7	548.5
Corn	11.5	12.6	Wheat flour	21.3	17.3	Corn gluten	677.7	598.6
Corn	12.0	19.3	Corn gluten meal	2.7	3.2	Feed	7.2	6.3
Corn	12.6	13.3	Corn gluten	3.1	3.7	Feed	11.3	10.9
Corn	13.9	15.3	Corn gluten meal	3.1	3.6	Feed	16.7	15.3
Corn	14.6	14.9	Corn gluten	5.6	4.5	Feed	20.3	23.5
Corn	15.1	12.6	Soybean meal	11.4	11.3	Feed	24.7	24.0
Corn	15.6	17.0	Soybean meal	11.4	12.3	Feed	28.8	29.0
Corn	16.7	18.9	Corn gluten meal	12.5	12.8	Feed	35.4	35.5
Corn	18.4	19.2	Corn gluten meal	13.2	16.3	Feed	42.7	43.3
Corn	19.4	17.6	Corn gluten	16.3	14.7	Feed	47.8	52.9
Corn	25.3	22.9	Corn gluten meal	19.4	36.4	Feed	59.6	56.8
Corn	25.9	31.9	Corn gluten	21.0	21.3	DDGS	63.0	77.7
Corn	35.7	38.9	Corn gluten meal	22.5	20.4	DDGS	70.4	117.8
Corn	54.2	47.9	Corn gluten meal	27.2	26.4	Feed	77.3	71.8
Corn	184.4	247.9	Corn gluten meal	28.1	25.5	Feed	77.3	93.5
Corn	743.2	750.5	Corn gluten	35.5	36.5	Feed	91.8	83.0
Wheat	3.0	3.9	Peanut meal	36.9	36.4	Feed	151.5	132.4

Wheat	4.3	3.8	Peanut meal	41.7	47.6	DDGS	153.9	158.9
Wheat	6.3	7.1	Corn gluten	42.5	35.4	Feed	163.0	119.1
Wheat	7.0	8.0	Peanut meal	45.7	59.9	Feed	184.0	196.5
Wheat	15.5	12.8	Corn germ	52.4	49.1	Feed	196.0	184.7
Wheat	36.4	49.8	Corn germ	56.7	56.5	DDGS	202.9	290.0
Wheat	117.5	123.3	Corn gluten meal	60.7	72.6	DDGS	245.7	205.5
Wheat flour	6.8	6.4	Corn gluten meal	65.4	69.1	Feed	526.8	497.4
Wheat flour	7.1	8.9	Corn germ	67.2	73.4	Feed	567.0	497.0
Wheat flour	7.2	4.4	Corn germ	67.2	74.3	Feed	567.0	526.3
Wheat flour	8.6	7.2	Corn germ	67.4	69.4	Feed	867.0	761.7

Table S3. The information of the authentic samples from China in 2019.

Number	Province	Sample	Number	Province	Sample	Number	Province	Sample
1	Jiangsu	Corn	33	Jiangsu	Corn germ	65	Anhui	DDGS
2	Jiangsu	Corn	34	Jiangsu	Corn germ	66	Anhui	DDGS
3	Jiangsu	Corn	35	Jiangsu	Corn germ	67	Shandong	Corn
4	Jiangsu	Corn	36	Jiangsu	Corn gluten	68	Shandong	Corn
5	Jiangsu	Corn	37	Jiangsu	Corn gluten	69	Shandong	Corn
6	Jiangsu	Corn	38	Jiangsu	Corn gluten	70	Shandong	Corn
7	Jiangsu	Corn	39	Jiangsu	Corn gluten	71	Shandong	Corn
8	Jiangsu	Corn	40	Jiangsu	Corn gluten meal	72	Shandong	Wheat
9	Jiangsu	Corn	41	Jiangsu	Peanut meal	73	Shandong	Wheat
10	Jiangsu	Wheat	42	Jiangsu	Peanut meal	74	Shandong	Wheat
11	Jiangsu	Wheat	43	Jiangsu	Peanut meal	75	Shandong	Wheat
12	Jiangsu	Wheat	44	Jiangsu	Feed	76	Shandong	Wheat flour
13	Jiangsu	Wheat	45	Jiangsu	Feed	77	Shandong	Wheat flour
14	Jiangsu	Wheat	46	Jiangsu	DDGS	78	Shandong	Corn gluten meal
15	Jiangsu	Wheat	47	Jiangsu	DDGS	79	Shandong	Corn gluten meal
16	Jiangsu	Wheat	48	Jiangsu	DDGS	80	Shandong	Corn germ
17	Jiangsu	Wheat	49	Anhui	Corn	81	Shandong	Corn gluten
18	Jiangsu	Wheat flour	50	Anhui	Wheat flour	82	Shandong	Corn gluten
19	Jiangsu	Wheat flour	51	Anhui	Wheat flour	83	Shandong	Rice bran meal
20	Jiangsu	Wheat flour	52	Anhui	Corn germ	84	Shandong	Rice bran meal
21	Jiangsu	Wheat flour	53	Anhui	Corn germ	85	Shandong	Soybean meal
22	Jiangsu	Wheat flour	54	Anhui	Corn germ	86	Shandong	Feed
23	Jiangsu	Wheat flour	55	Anhui	Rice bran meal	87	Hebei	Corn
24	Jiangsu	Wheat flour	56	Anhui	Rice bran meal	88	Hebei	Corn
25	Jiangsu	Wheat flour	57	Anhui	Peanut meal	89	Hebei	Corn
26	Jiangsu	Wheat flour	58	Anhui	Peanut meal	90	Hebei	Corn
27	Jiangsu	Wheat flour	59	Anhui	Peanut meal	91	Hebei	Corn
28	Jiangsu	Wheat flour	60	Anhui	Peanut meal	92	Hebei	Wheat flour
29	Jiangsu	Wheat flour	61	Anhui	Soybean meal	93	Hebei	Wheat flour
30	Jiangsu	Wheat flour	62	Anhui	Soybean meal	94	Hebei	Wheat flour
31	Jiangsu	Wheat flour	63	Anhui	Feed	95	Henan	Corn

32	Jiangsu	Wheat flour	64	Anhui	DDGS	96	Henan	Corn

Table S4. The information of the authentic samples from China in 2019.

Number	Province	Sample	Number	Province	Sample	Number	Province	Sample
97	Henan	Corn	128	Liaoning	Wheat flour	159	Jiangxi	Corn
98	Henan	Corn	129	Liaoning	Wheat flour	160	Jiangxi	Corn germ
99	Henan	Wheat	130	Liaoning	Wheat flour	161	Zhejiang	Corn
100	Henan	Wheat	131	Liaoning	Wheat flour	162	Zhejiang	Corn
101	Henan	Wheat	132	Liaoning	Corn gluten meal	163	Zhejiang	Wheat flour
102	Henan	Wheat	133	Liaoning	Soybean meal	164	Zhejiang	Corn germ
103	Henan	Feed	134	Liaoning	Peanut meal	165	Zhejiang	Corn gluten meal
104	Henan	Feed	135	Liaoning	Feed	166	Zhejiang	Rice bran meal
105	Henan	Feed	136	Liaoning	Feed	167	Zhejiang	Soybean meal
106	Neimenggu	Corn	137	Liaoning	Feed	168	Zhejiang	Feed
107	Neimenggu	Corn	138	Liaoning	Feed	169	Zhejiang	Feed
108	Neimenggu	Corn	139	Liaoning	DDGS	170	Zhejiang	Feed
109	Neimenggu	Wheat	140	Liaoning	Wheat flour	171	Zhejiang	Feed
110	Neimenggu	Wheat	141	Heilongjiang	Corn	172	Hunan	Wheat flour
111	Neimenggu	Wheat flour	142	Heilongjiang	Wheat	173	Hunan	Wheat flour
112	Neimenggu	Wheat flour	143	Heilongjiang	Corn germ	174	Hunan	Wheat flour
113	Neimenggu	Wheat flour	144	Heilongjiang	Feed	175	Hunan	Wheat flour
114	Neimenggu	Wheat flour	145	Heilongjiang	Feed	176	Hunan	Corn germ
115	Neimenggu	Corn germ	146	Heilongjiang	Feed	177	Hunan	Corn germ
116	Neimenggu	Peanut meal	147	Heilongjiang	DDGS	178	Hunan	Corn gluten meal
117	Neimenggu	Soybean meal	148	Heilongjiang	DDGS	179	Hunan	Corn gluten meal
118	Neimenggu	Feed	149	Heilongjiang	DDGS	180	Hunan	Rice bran meal
119	Neimenggu	Feed	150	Jiangxi	Corn	181	Sichuan	Wheat flour
120	Neimenggu	Feed	151	Jiangxi	Corn	182	Sichuan	Wheat flour
121	Neimenggu	Feed	152	Jiangxi	Corn	183	Sichuan	Wheat flour
122	Neimenggu	Feed	153	Jiangxi	Corn	184	Sichuan	Corn gluten meal
123	Neimenggu	Feed	154	Jiangxi	Corn	185	Sichuan	Feed
124	Neimenggu	Feed	155	Jiangxi	Corn	186	Guangxi	Corn gluten meal
125	Neimenggu	Feed	156	Jiangxi	Corn	187	Guangxi	Feed

126	Neimenggu	Feed	157	Jiangxi	Corn
127	Liaoning	Wheat flour	158	Jiangxi	Corn

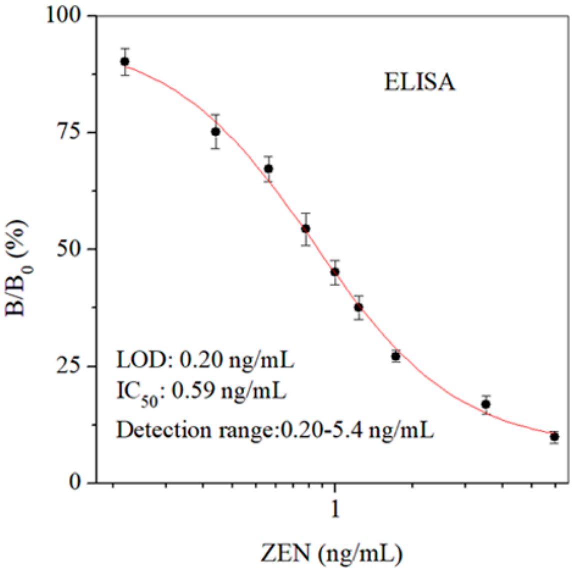

Figure S1. The standard curve of ELISA for ZEN.

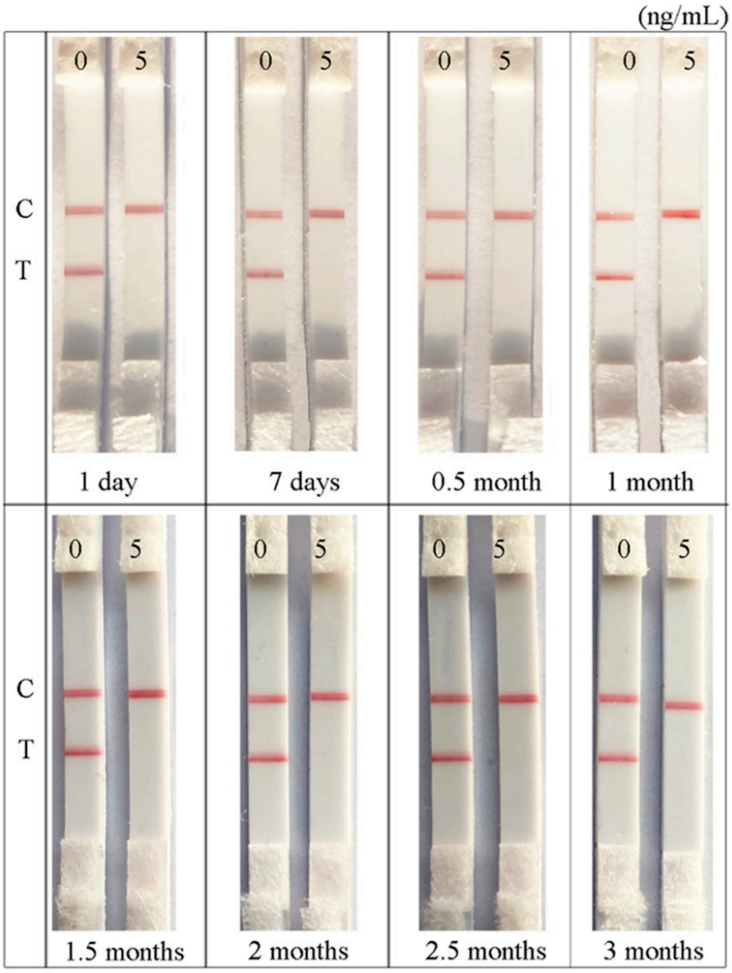

Figure S2. The stability and sensitivity tests of the ICA strip for ZEN in the storage of 3 months.

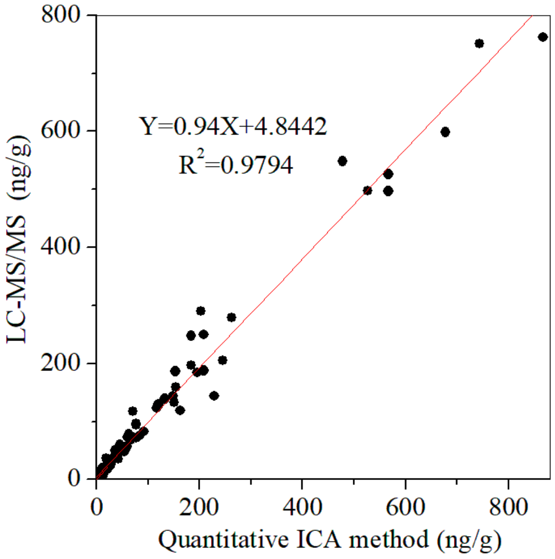

Figure S3. The correlation between the quantitative ICA and LC-MS/MS for ZEN in the authentic samples.
